# Protective effects of phenethyl isothiocyanate on foam cell formation by combined treatment of oxidized low‐density lipoprotein and lipopolysaccharide in THP‐1 macrophage

**DOI:** 10.1002/fsn3.2293

**Published:** 2021-05-04

**Authors:** Young‐Sun Im, Min‐Hee Gwon, Jung‐Mi Yun

**Affiliations:** ^1^ Department of Food and Nutrition Chonnam National University Gwangju Korea; ^2^ Nutrition Education Major Graduate School of Education Chonnam National University Gwangju Korea

**Keywords:** atherosclerosis, foam cells, lipopolysaccharides, oxidized low density lipoprotein, phenethyl isothiocyanate, scavenger receptor

## Abstract

Accumulation of cholesterol‐laden macrophage foam cells characteristic of early stage atherosclerotic lesions. Phenethyl isothiocyanate (PEITC) is a naturally occurring isothiocyanate found in cruciferous vegetables that has reported a variety of activities including antioxidant and anti‐inflammatory properties. However, the protective effect of PEITC on foam cell formation and its precise mechanism is not yet clear. Therefore, we investigated whether PEITC suppresses foam cell formation and regulates the expression of genes related to lipid accumulation, cholesterol efflux, and inflammation in THP‐1 derived‐macrophages. We exposed THP‐1 derived‐macrophages to oxidized low‐density lipoprotein (ox‐LDL) (20 μg/mL) and lipopolysaccharide (LPS) (500 ng/ml) to mimic foam cell formation. Here, PEITC downregulated the expression of lectin‐like oxidized low‐density lipoprotein receptor‐1 (LOX‐1), cluster of differentiation 36 (CD36), scavenger receptor A1 (SR‐A1), and nuclear factor‐κB (NF‐κB), while upregulated ATP binding cassette subfamily A member 1 (ABCA1)/liver‐X‐receptor α (LXR‐α)/peroxisome proliferator‐activated receptor gamma (PPARγ) and sirtuin 1 (SIRT1) expression compared to co‐treated with ox‐LDL and LPS. Taken together, PEITC, at least in part, inhibits foam cell formation and reduces lipid accumulation in foam cells. Therefore, we suggest that PEITC may be a potential candidate for the treatment and prevention of vascular inflammation and atherosclerosis.

## Graphical abstract

PEITC control foam cell formation by promoting cholesterol efflux, reducing lipid accumulation, and modulating the SIRT1‐NF‐κB signaling pathway.

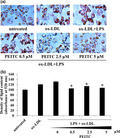

## 1 INTRODUCTION

Atherosclerosis is the dominant cause of cardiovascular disease (CVD), resulting in a high rate of mortality in humans. Nutrition is an important to human health in the context of preventing the development of chronic diseases such as cancer and atherosclerosis ("Diet, nutrition and the prevention of chronic diseases," 2003). Nutrition strategies are important for handling metabolic disorders, such as CVD and atherosclerosis. Many phytochemicals, including fiber, vitamins, minerals, and antioxidants, are found in fruits and vegetables (Liu, 2013). These phytochemicals have demonstrated a clear effect on the apparent reduction in CVD risk reduction either independently or by synergistic effects with other nutrients (Pop et al., 2018).

Cholesterol accumulation in monocyte‐derived macrophages is converted to foam cells that comprise the characteristic fat streaks observed in the early stages of atherosclerosis (Maguire et al., 2019; Rafieian‐Kopaei et al., 2014). During atherosclerosis, when lipids gradually accumulate in the subcutaneous space of the damaged artery, macrophages are absorbed from the arterial wall. Furthermore, increasing cholesterol levels in blood and vascular inflammation are general characteristics of atherosclerosis (Chuang et al., 2012).

In the early stage of atherosclerosis, macrophage foam cells are primarily formed by uncontrolled absorption of oxidized low‐density lipoproteins (ox‐LDL), excessive cholesterol esterification, and/or impaired cholesterol efflux, resulting in the accumulation of cholesterol esters as cytoplasmic lipid droplets (Chistiakov et al., 2016; Ouimet et al., 2011; Yu et al., 2013).

In atherosclerosis progression, atherosclerotic plaques develop upon accumulation of foam cells derived from macrophages that take up ox‐LDL. Scavenger receptors (SRs), including scavenger receptor class A1 (SR‐A1), cluster of differentiation 36 (CD36), and lectin‐like oxidized low‐density lipoprotein receptor‐1 (LOX‐1) are the principal receptors responsible for the binding and uptake of oxidized low‐density lipoprotein (ox‐LDL) in macrophages (Levitan et al., 2010). Lipopolysaccharide (LPS) enhances the expression of SRs and thus increases the uptake of ox‐LDL into macrophages (Hashimoto et al., 2020). The ATP‐binding cassette (ABC) transporter A1(ABCA1), ABCG1, and scavenger receptor‐B1 (SR‐B1) are three important mediators of cholesterol efflux from macrophages (Back et al., 2019). Among them, ABCA1 promotes cholesterol efflux from cells to lipid‐poor apolipoprotein A‐I (Xu et al., 2011). Liver X receptor alpha (LXR‐α) and peroxisome proliferator‐activated receptor gamma (PPARγ) act as cholesterol sensors that may protect from cholesterol overload by stimulating cholesterol efflux from cells to high‐density lipoproteins via ABCA1 (Ma et al., 2015). Thus, the ABCA1/LXR‐α/PPARγ interaction is essential for cholesterol homeostasis (Ogata et al., 2009; Soumian et al., 2005).

Chronic inflammation induces the release of pro‐inflammatory cytokines such as tumor necrosis factor‐α (TNF‐α), monocyte chemoattractant protein‐1 (MCP‐1), and interleukin 6 (IL‐6); immune cell infiltration is closely related to the pathogenesis of atherosclerosis in vessel walls (Ross, 1999). Nuclear factor‐κB (NF‐κB) transcription factor is one of the major regulators of inflammation and is involved in the onset and progression of pathogenesis in atherosclerosis (Grote et al., 2005). Moreover, LPS exacerbates atherosclerosis by inducing the upregulation of NF‐κB, enhancing the expression of scavenger receptors and thus increasing the uptake of ox‐LDL into macrophages (Hashimoto et al., 2020). ox‐LDL induces the dysregulation of NF‐κB and is potentially involved in atherosclerosis (Robbesyn et al., 2004). Sirtuin 1 (SIRT1) plays an additional protective role in vascular biology and atherosclerosis (Stein & Matter, 2011). It has anti‐inflammatory functions that disrupt the NF‐κB signaling pathway, downregulating the expression of various pro‐inflammatory cytokines in endothelial cells and macrophages (Feng et al., 2018). A previous study reported that SIRT1 overexpression in transgenic mice inhibited NF‐κB activity induced by high‐fat foods and reduced specific pro‐inflammatory cytokines, including TNFα and IL‐6, promoting fatty liver (Pfluger et al., 2008).

Thus, inhibition of foam cell formation, inflammation, and stimulation of cholesterol efflux may be an effective approach to prevent the progression of atherosclerotic lesions.

A previous study reported that high intake of vegetables, especially cruciferous, is important in reducing the risk of cancer in diverse ways, due to effects including (a) metabolic activation and detoxification, (b) inflammation, (c) angiogenesis, (d) metastasis, and (e) regulation of the epigenetic machinery (Mitsiogianni et al., 2019). Phenethyl isothiocyanate (PEITC) is formed upon hydrolysis of the parent glucosinolates, which occurs in cruciferous vegetables including cabbage, kale, broccoli, and Brussels sprouts (Park et al., 2017). In vivo and in vitro studies have shown that PEITC regulates cancer‐related gene expression, such as antioxidant response, resistance to apoptosis, cell cycle regulation, and metastasis (Chou et al., 2018; Lam‐Ubol et al., 2018). Furthermore, in our previous study, we showed that PEITC supplementation in high fat/cholesterol diet‐fed mice reduced fat/cholesterol accumulation in the liver and aorta and decreased liver HFCD‐related inflammation (Gwon et al., 2020). Although, the protective effects of PEITC on foam cell formation and the molecular mechanisms that regulate cholesterol efflux upregulation of ABCA1 expression by treatment with PEITC in macrophage foam cells are not understood.

Our study investigated the anti‐foam cell formation effect of PEITC and attempted to identify the pathways involved using THP‐1‐derived foam cells to establish in vitro early stage atherosclerosis models, which represent inflammatory and lipid‐laden macrophages. Furthermore, we analyzed the effect of PEITC supplementation on the expression of genes related to lipid accumulation, cholesterol efflux, and inflammation.

## 2 MATERIALS AND METHODS

### 2.1 Materials

PEITC (GC ≥ 99%) was procured from Sigma Aldrich. LPS, phorbol 12‐myristate 13‐acetate (PMA), and thiazolyl blue tetrazolium bromide (MTT) were procured from Sigma‐Aldrich. IL‐6 and TNF‐α enzyme‐linked immunosorbent assay (ELISA) kits were procured from Raybio. The BCA protein assay kit and ox‐LDL were procured from Thermo Fisher Scientific. Unless otherwise stated, all other chemicals were procured from Sigma Aldrich or Biosesang.

### 2.2 THP‐1 cell culture and PMA‐induced differentiation

Human THP‐1 cells were obtained from the Korean Cell Line Bank (KCLB, Seoul, South Korea). Cells were cultured in RPMI‐1640 medium containing 10% fetal bovine serum (FBS) and 1% antibiotics (Welgene) and incubated at 37°C with 5% CO_2_. Cells between passages 5 and 10 were used for the experiments. THP‐1 cells (1 × 10^6^ cells/mL) were differentiated into macrophages by stimulation with a PMA concentration of 1 μM for 48 hr. Differentiated THP‐1 cells were cultured for 48 hr in the presence or absence of PEITC at various concentrations and treated with 20 μg/mL ox‐LDL and 500 ng/ml LPS for 24 hr prior to harvest. Before harvesting the cells, the culture medium for cytokine secretion measurement was collected, and the cells were washed twice with phosphate‐buffered saline (PBS), and then harvested.

### 2.3 Measurement of cell viability

The cytotoxicity of PEITC on PMA‐activated THP‐1 macrophages was measured using the MTT assay. The cells were seeded at a concentration of 1 × 10^6^ cells/well in 24‐well plates and treated with PEITC for 48 hr and treated with LPS (500 ng/ml) for 6 hr prior to harvest, and MTT solution (100 μL; 1 mg/ml) was added and incubated for a further 2 hr. The precipitated formazan was solubilized in 1 ml of 100% dimethyl sulfoxide. Finally, plates were placed in an EZRead 400 microplate reader (Biochrom) to measure absorbance at 570 nm.

### 2.4 Oil Red O staining

Cells were examined for lipid accumulation by Oil Red O staining. Briefly, cells were incubated with 10% formalin (Sigma‐Aldrich) for 30 min at 4°C and then treated with 0.5% Oil Red O solution (Sigma‐Aldrich) dissolved in 60% isopropanol for 30 min. Images were collected using a Leica microscope and Leica Application Suite X software (Leica Microsystems, Wetzlar, Germany). A 400× objective was used for all images. The degree of staining was quantified by measuring absorbance at 520 nm using an EZRead 400 microplate reader.

### 2.5 ELISA

To analyze the effect of PEITC on cytokine production under THP‐1‐derived macrophages exposed to ox‐LDL (20 μg/mL) and LPS (500 ng/ml), the cells were seeded at a concentration of 1 × 10^6^ cells/mL/well in 24‐well plates, treated with PEITC at various concentrations (0.5–5 μM) for 24 hr, and then LPS (500 ng/ml) for 24 hr without media change before forming foam cells. Cell‐free supernatants were collected and stored at −20°C until they were assayed for cytokine levels using an ELISA kit.

### 2.6 Immunoblotting

For immunoblotting, 10–30 μg protein was separated on 8%–12% polyacrylamide gels and transferred to nitrocellulose membrane (Merck Millipore Ltd., Tullagreen, Carrigtwohill, County Cork, Ireland). The blot was blocked and reacted 2 hr at 4°C with each antibody against ABCA1, LOX‐1, SR‐A1, CD36, PPARγ, LXR‐α, SIRT1, and NF‐κB. Proteins were detected with the appropriate secondary antibodies and visualized by applying the Western Blotting Luminol Reagent (Santa Cruz Biotechnology). Finally imaged on a ChemiDoc XRS + System (Bio‐Rad). The blots were stripped and then reprobed with β‐actin and Histone H1 as loading controls.

### 2.7 Immunofluorescence

After treatment with PEITC, cells were washed twice in PBS, fixed with 4% formaldehyde for 30 min at 4°C, and stained overnight with NF‐κB antibody (1:100 dilution). After air drying, slides were incubated with secondary antibody for 60 min. The nuclei were stained with DAPI (100 ng/ml, Beyotime Biotechnology), and the samples were washed thrice with PBS. DAPI solution was incubated at 37°C for nucleic acid detection. The slides were washed twice in PBS, air‐dried, treated with mounting medium, and then examined under a fluorescence microscope at 400× magnification. Images were collected using the Leica Application Suite X software. The signal intensity was analyzed using Image J software.

### 2.8 Quantitative Polymerase Chain Reaction (qPCR)

Total RNA was prepared from cultured cells using TRIzol reagent (Thermo Fisher Scientific). cDNA was synthesized from total RNA through reverse transcription using the cDNA synthesis kit (Omniscript RT kit) with 1 μg RNA after confirming the RNA concentration and purity assessment. SYBR green‐based qPCR was performed with the CFX96 Touch Real‐Time PCR Detection System (Bio‐Rad) and iQ SYBR Green Supermix (Bio‐Rad). qPCR was performed using the following primers (5′ to 3′, Forward: F; Reverse: R): human ABCA1 (F): GTCCTCTTTCCCGCATTATCTGG (R): AGTTCCTGGAAGGTCTTGTTCAC, human PPARγ (F): CACAAGAACAGATCCAGTGGTTGCAG (R): AATAATAAGGTGGAGATGCAGGCTCC, human LXR‐α (F): ACACCTACATGCGTCGCAAG (R): GACGAGCTTCTCGATCATGCC, human LOX‐1 (F): GGGCTCATTTAACTGGGAAA (R): GAAATTGCTTGCTGGATGAA, human CD36 (F): GGGAAAGTCACTGCGACATG (R): TGCAATACCTGGCTTTTCTCA, human β‐actin (F): ACCCCGTGCTGCTGAC (R): CCAGAGGCGTACAGGGATAG. Results were normalized using β‐actin as a housekeeping gene and evaluated using the 2^−ΔΔCt^ method.

### 2.9 Statistical analysis

Each experiment was repeated three times, and data are expressed as means ± standard deviation (*SD*). Statistical analyses were conducted with one‐way ANOVA followed by Dunnett's post hoc test (SPSS version 25.0 software, SPSS Institute). Significance was defined as *p* < .05 or *p* < .01, and specific significance values are stated in the figure legends.

## 3 RESULTS

### 3.1 Co‐treatment of ox‐LDL and LPS accelerates foam cells in THP‐1 macrophages

**FIGURE 1:**
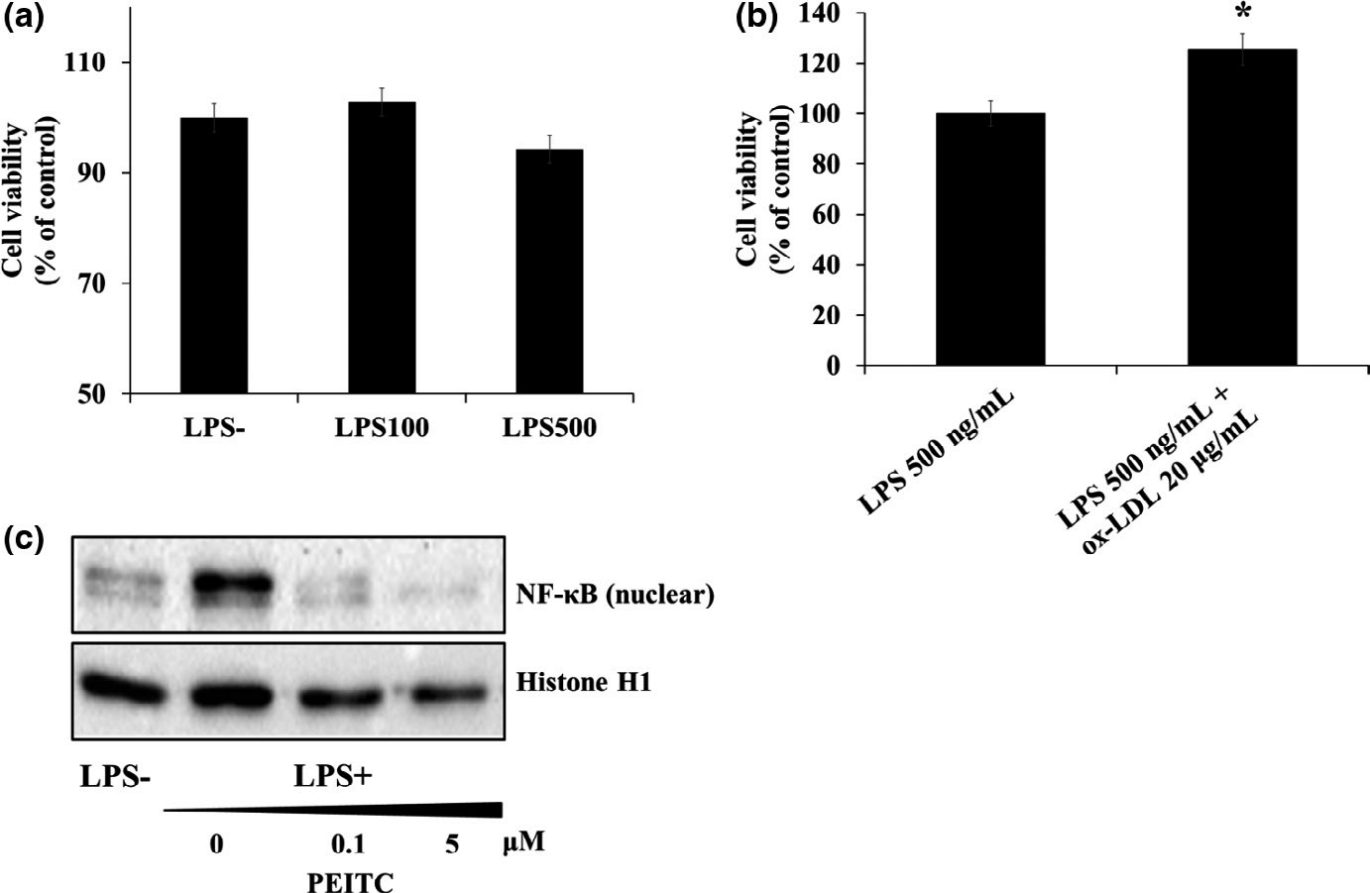
Effect of LPS (500 ng/ml) and ox‐LDL (20 μg/ml) on cell viability of THP‐1 foam cells and upregulated expression of inflammatory factor. THP‐1 monocytes were exposed to 1 μM of phorbol 12‐myristate 13‐acetate (PMA) for 48 hr and incubated for 24 hr with two ox‐LDL concentrations (10 or 20 μg/mL). Relative cell viability was determined by the amount of MTT converted into formazan crystals and quantified as a percentage of control. The data are presented as the mean ± *SD* (*n* = 3 independent experiments). (a) The cytotoxicity of LPS in THP‐1 cells. (b) The cytotoxicity of ox‐LDL in presence of LPS (500 ng/ml) THP‐1 cells. (c) The NF‐κB expression in nucleus. *p < .05 versus LPS

THP‐1 cells are notably sensitive to LPS and respond by expressing many inflammatory cytokines. Thus, we used LPS to induce acute inflammation. To determine the noncytotoxicity concentration of LPS to the cells, we used MTT assay. The results showed that there was no cytotoxicity at 100 and 500 ng/ml (Figure 1a) in THP‐1 macrophages, so we used concentration of LPS 500 ng/ml in this experiment. Foam cell formation from macrophages is essential for the progression of atherosclerotic lesions. In acute inflammation, ox‐LDL accelerates the formation of foam cells (Kruth, 2001). To determine the concentration of ox‐LDL that forms foam cells in differentiated THP‐1 cells but does not cause toxicity, cytotoxicity was first confirmed by simultaneously treating cells with LPS and ox‐LDL. ox‐LDL treatment at a concentration of 20 μg/mL showed no toxicity compared with that by only LPS at 500 ng/ml in THP‐1 macrophages (*p* < .05).

Before identifying the protective effect of PEITC for the treatment of LPS, the following experiment was conducted to establish the concentrations to be used in the experiments of PEITC. There was no cytotoxic effect of PEITC at 0–5 μM on LPS‐induced THP‐1 macrophages (*p* < .05) (data not shown). In addition, as shown in 1C, PEITC reduced NF‐κB expression in LPS‐induced THP‐1 macrophages.

**FIGURE 2:**
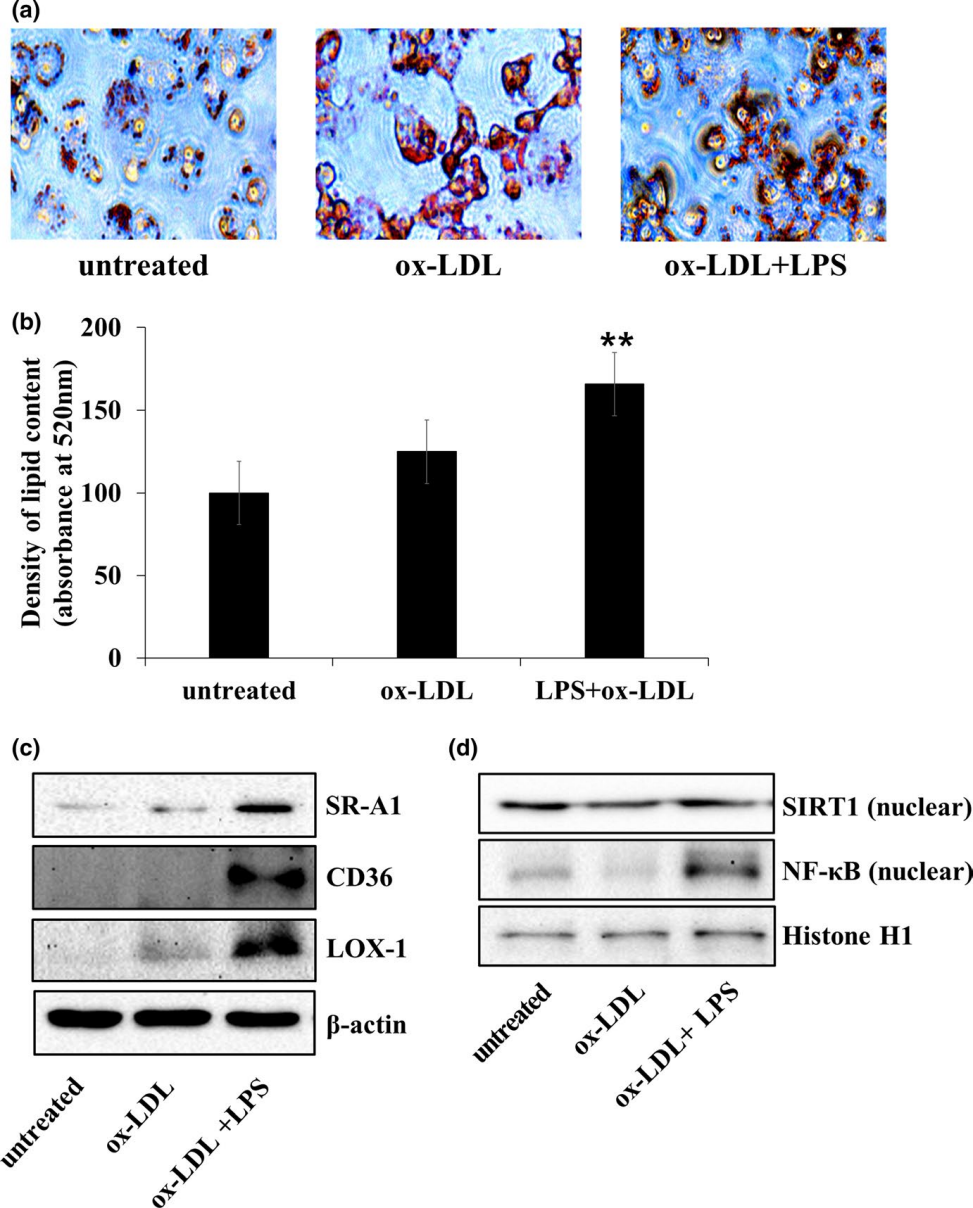
Acceleration of foam cell formation by ox‐LDL and LPS co‐treatment in THP‐1 foam cells. (a) Foam cell formation was determined by Oil Red O staining. The degree of staining was quantified by measuring absorbance at 520 nm; magnification 400×. (b) Stained cells were dissolved in isopropanol solution and the staining intensity was measured at 520 nm. Oil Red O areas were calculated as percentages of the stained cell area to the total cell area. The data are presented as the mean ± *SD* (*n* = 3 independent experiments). **p < .01 versus untreated cells. (c) Cell lysates were prepared for immunoblotting with each antibody as lipid receptor protein. β‐actin was used as an internal control. (d) SIRT1 and NF‐κB protein expression in foam cells in nucleus. Histone H1 was used as an internal control

Treatment ox‐LDL 20 μg/mL increased lipid accumulation compared with untreated ox‐LDL, but there was no significant difference (Figure 2a,b). And we observed the strongest red staining in foam cells when ox‐LDL and LPS were co‐treated, indicating that cellular accumulation of lipids occurred under these conditions (*p* < .01). Therefore, co‐treated 20 μg/mL of ox‐LDL and 500 ng/ml of LPS was considered to be an optimal concentration in vitro environment for carrying out this experiment.

In addition, when cells were co‐treated with ox‐LDL and LPS for 24 hr, the expression levels of SR‐A1, CD36, and LOX‐1 were upregulated (Figure 2c). As shown in Figure 2d, SIRT1 expression was slightly decreased and NF‐κB expression increased upon co‐treatment with ox‐LDL and LPS. Thus, co‐treatment with ox‐LDL and LPS causes more inflammation than only ox‐LDL treatment.

### 3.2 PEITC inhibits foam cell formation via downregulating cellular lipid accumulation

**FIGURE 3:**
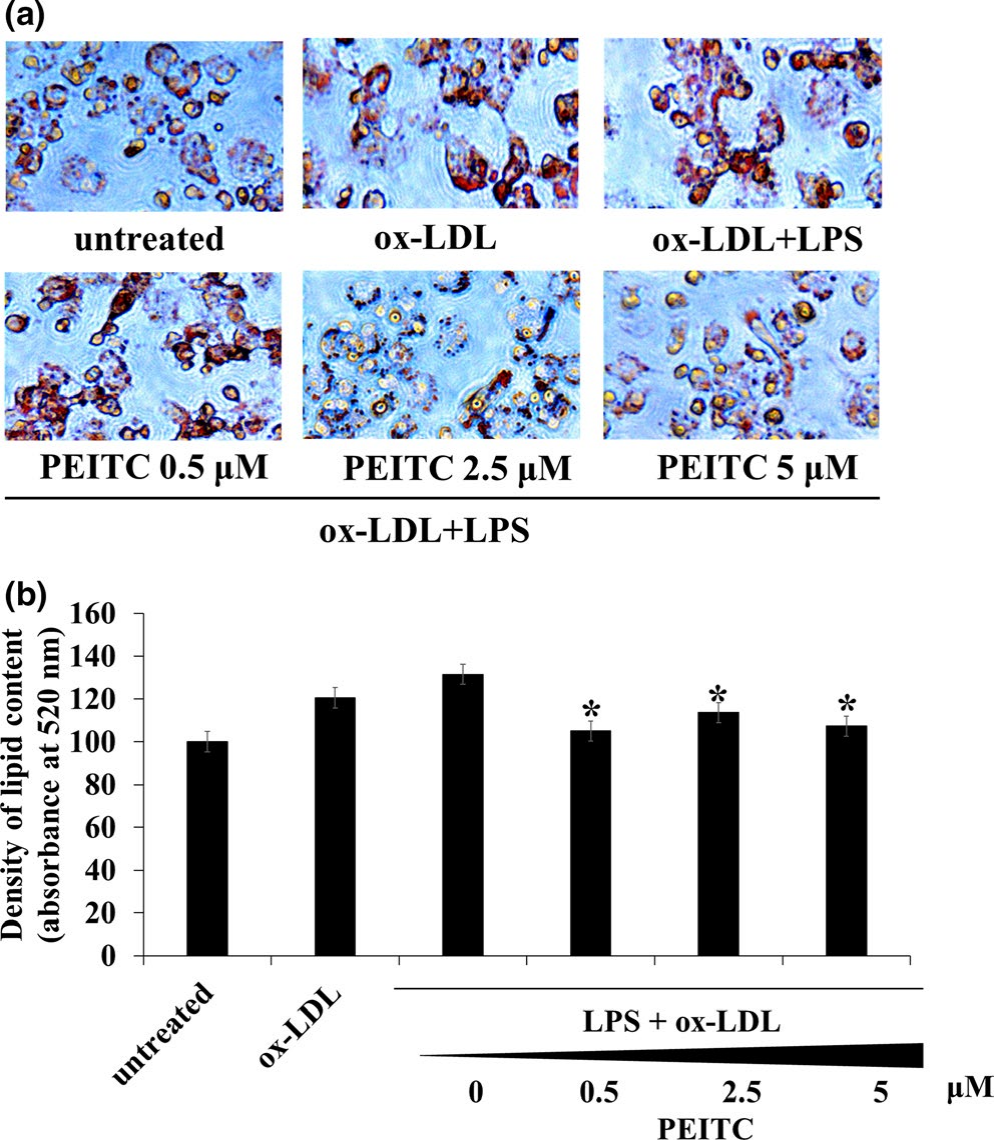
Downregulation of lipid accumulation by PEITC treatment in THP‐1 foam cells. THP‐1 differentiated macrophages were cultured in the absence or presence of PEITC (0– 5 μM) prior to 24 hr. Then, THP‐1 cell was cultured in LPS (500 ng/ml) containing ox‐LDL (20 μg/ml) for 24 hr. (a) Cells were stained with Oil Red O, microphotographs were obtained using an optical microscope, magnification 400×. (b) Stained cells were treated with isopropanol solution and the staining intensity was measured at 520 nm. Oil Red O areas were calculated as percentages of the stained cell area to the total cell area. The data are presented as the mean ± *SD* (*n* = 3 independent experiments). *p < .05 versus co‐treated ox‐LDL and LPS without PEITC

We examined the effect of PEITC on the degree of inhibition of lipid accumulation in foam cells using Oil Red O staining and immunoblotting. As shown in Figure 3, there was strong red staining was observed in foam cells exposed to 20 μg/mL ox‐LDL and LPS for 24 hr. This suggested that cellular accumulation of lipids was significantly upregulated by co‐treatment with ox‐LDL and LPS compared to that in untreated cells. However, this strong red stain was downregulated by treatment with PEITC for 48 hr. In addition, we quantified the intracellular lipid stained by Oil Red O at‐ 520 nm absorbance using spectrophotometry. Thus, PEITC has an inhibitory effect on lipid accumulation.

### 3.3 PEITC suppresses expression of lipid receptors in foam cells

**FIGURE 4:**
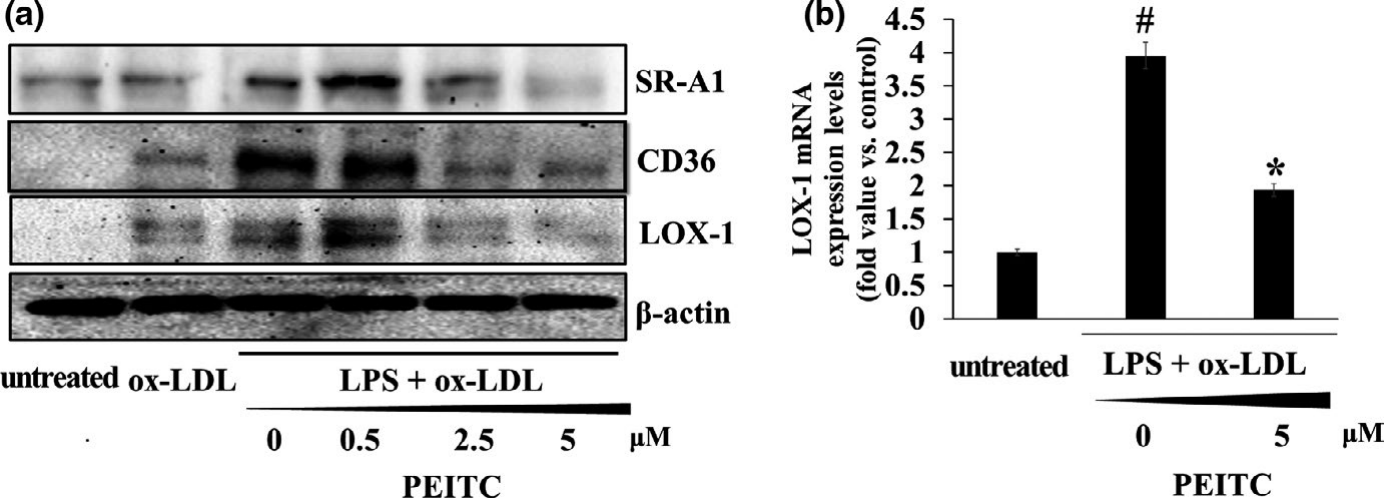
Inhibition of SR‐A1, CD36, and LOX‐1 expression by PEITC treatment in THP‐1 foam cells. (a) Immunoblotting was used to measure the protein levels of SR‐A1, CD36, and LOX‐1. β‐actin was used as an internal control. (b) mRNA levels of LOX‐1 were analyzed using qPCR. Significance was determined by comparison with β‐actin normalized 2^−∆∆CT^ values. Data represent the mean ± standard deviation (*SD*, *n* = 3 independent experiments)

We observed the effect of PEITC on cellular lipid uptake in foam cells. As shown in Figure 4, we measured the expression levels of the lipid uptake‐related proteins, LOX‐1, SR‐A1, and CD36. It was shown that when foam cells were treated with PEITC for 48 hr, the expression levels of lipid receptor proteins LOX‐1, CD36, and SR‐A1were reduced in a dose‐dependent manner. This demonstrated that PEITC inhibits the formation of foam cells by suppressing the expression of lipid receptors. In addition, the transcription levels of these proteins were investigated by using qPCR. The transcription of the ox‐LDL receptor mRNA LOX‐1 increased in lipid‐laden macrophages, but LOX‐1 mRNA was observed to decrease by PEITC treatment in foam cells.

### 3.4 PEITC increases expression of the cholesterol efflux proteins in foam cells

**FIGURE 5:**
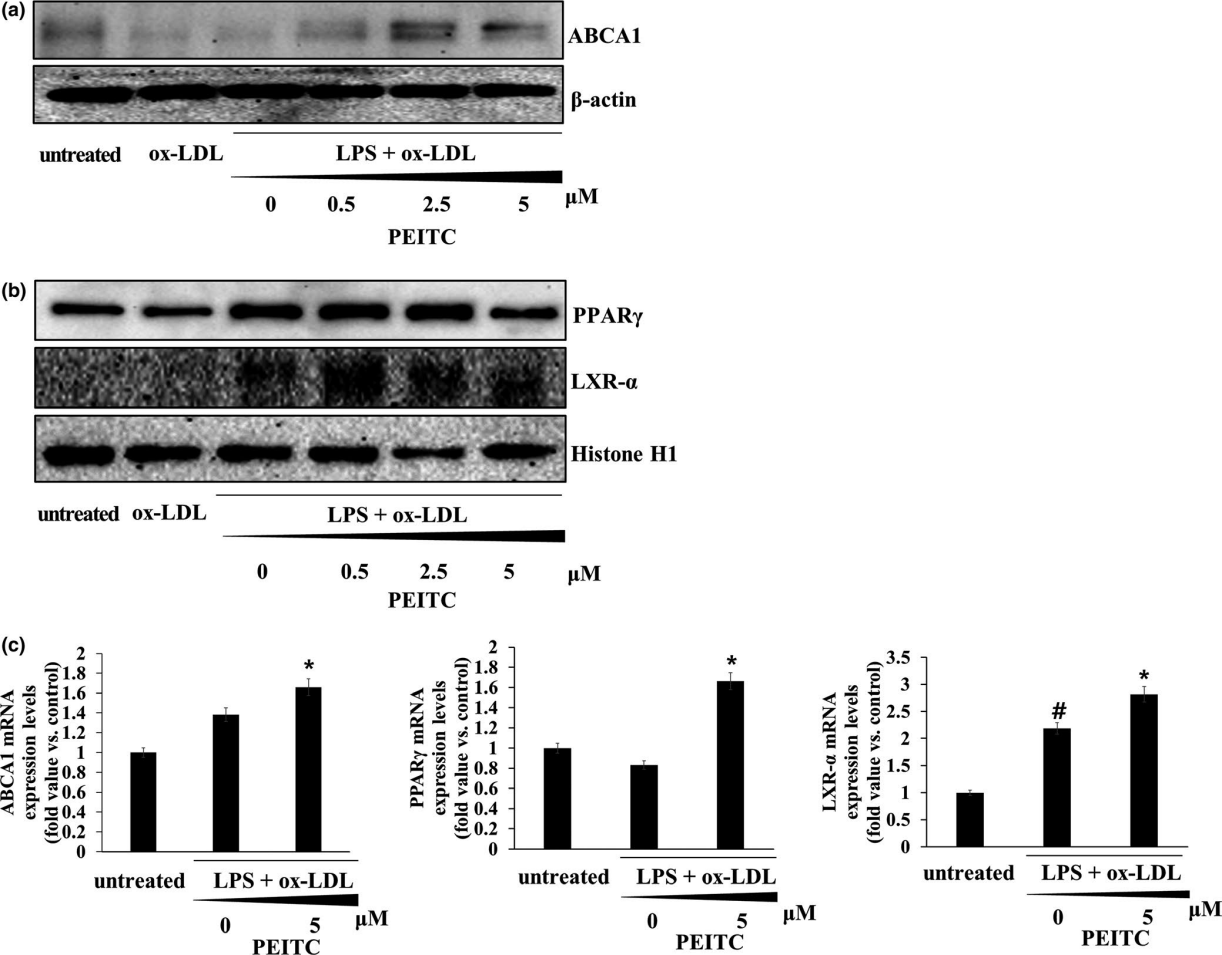
Upregulation of ABCA1, PPARγ, and LXR‐α expression by PEITC treatment in THP‐1 foam cells. (a) Immunoblotting was used to measure the protein levels of ABCA1. β‐actin was used as an internal control. (b) Immunoblotting was used to measure the protein levels of PPARγ/LXR‐α. Histone H1 was used as an internal control. (c) mRNA levels of ABCA1, PPARγ, and LXR‐α were analyzed using qPCR. Significance was determined by comparison with β‐actin normalized 2^−∆∆CT^ values. Data represent the mean ± standard deviation (*SD*, *n* = 3 independent experiments)

We examined the effect of PEITC on cellular cholesterol efflux in foam cell. As shown in Figure 5a, using immunoblotting, we measured the expression levels of ABCA1, the cellular cholesterol efflux protein. It was shown that when foam cells were treated with PEITC for 48 hr, the expression of ABCA1 decreased, but expression of ABCA1 increased at 2.5 μM and 5 μM of PEITC. These findings suggest that PEITC inhibits foam cell formation by promoting cholesterol efflux in cells.

Additionally, using immunoblotting, LXR‐α expression level was observed to be upregulated in the PETIC treated foam cells (Figure 5b). Previous studies have demonstrated that PPARγ and LXR‐α play pivotal roles in stimulating cholesterol efflux from macrophage foam cells (Chawla et al., 2001; Majdalawieh & Ro, 2010).

In addition, the transcription levels of these proteins were investigated using qPCR (Figure 5c). As expected, the transcription of ABCA1, PPARγ, and LXR‐α mRNA increased in PEITC treated‐foam cells. Therefore, PEITC is considered to promote ABCA1 transcription through the regulation of LXR‐α/PPARγ.

### 3.5 PEITC suppressed secretion of pro‐inflammatory cytokines and transactivation of NF‐κB in foam cells

**FIGURE 6:**
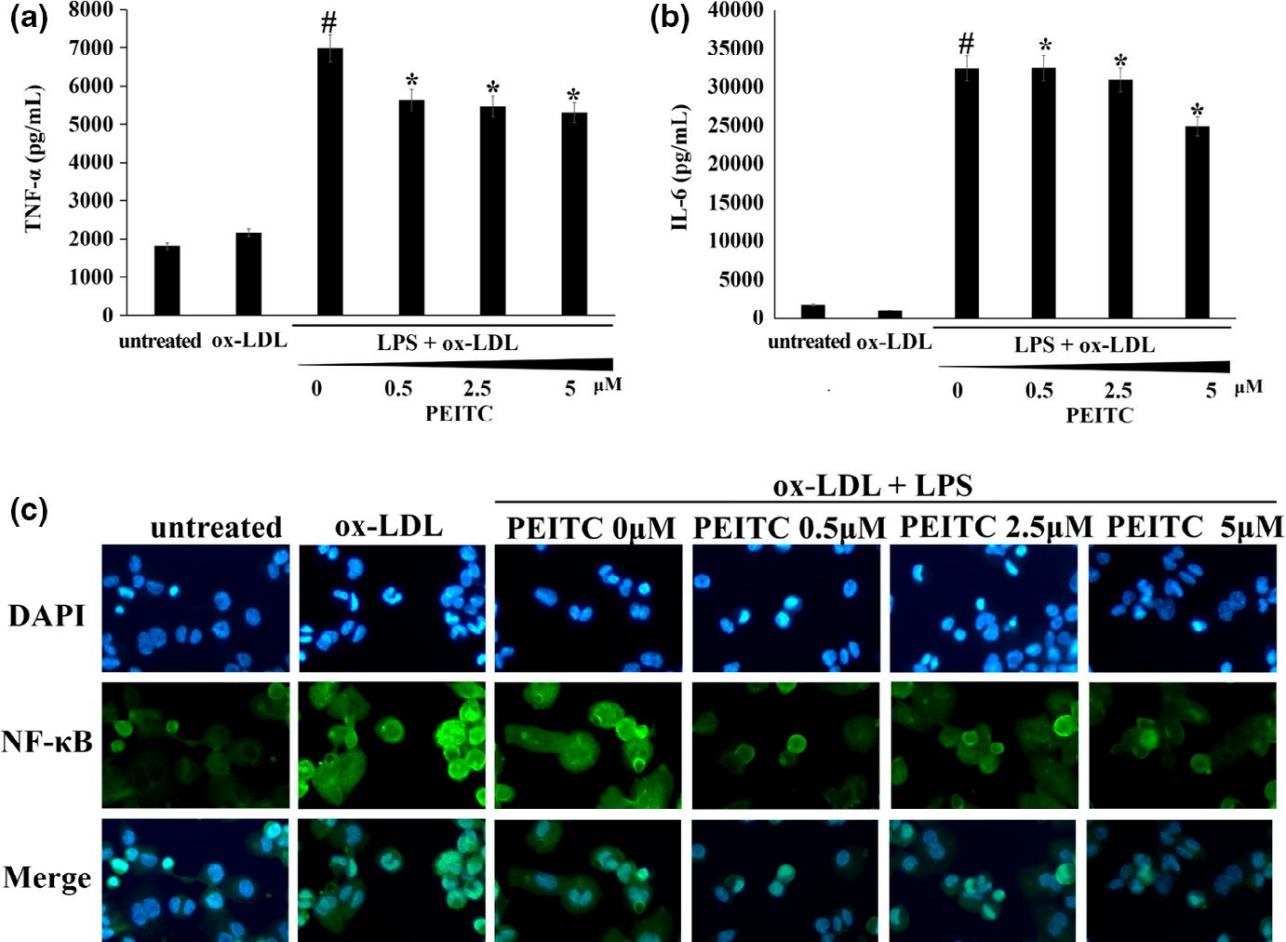
Inhibition of NF‐κB p65 activation and inflammatory cytokine secretion by PEITC treatment in co‐treated with ox‐LDL and LPS‐induced foam cell. THP‐1 foam cells were pretreated with different concentration of PEITC (0–5 μM) for 48 hr. Cell media were collected for IL‐6 and TNF‐α and measured using an ELISA kit. (a) PEITC reduced TNF‐α secretion in co‐treatment ox‐LDL and LPS. (b) PEITC reduced IL‐6 secretion in co‐treatment ox‐LDL and LPS. Data represent the mean ± standard deviation (*SD*, *n* = 3 independent experiments). #*p* < .05 versus untreated cell; *p < .05 versus co‐treated ox‐LDL and LPS without PEITC. (c) THP‐1 foam cells were fixed with 4% paraformaldehyde. After blocking, the cells were subsequently incubated with primary (NF‐κB) and secondary antibody. Signal quantification was assessed by fluorescence microscope; Magnification 400×

Pro‐inflammatory cytokines play a key role in vascular inflammation, leading to the development of atherosclerosis and coronary artery disease (Labinskyy et al., 2006; Li et al., 2005). As shown in Figure 6, NF‐κB has been reported to play a pivotal role in the inflammatory response through the induction of inflammation‐related cytokines (i.e., IL‐6, and TNF‐α). In our study, we identified the effect of PEITC on cytokine production in foam cells using ELISA. Co‐treatment with LPS and ox‐LDL significantly increased these cytokine levels compared with those in the untreated cells. However, these increases were significantly abrogated by PEITC.

In addition to cytokine secretion, we evaluated the effect of PEITC on NF‐κB activation in foam cells using immunofluorescence. As shown in Figure 6c, activation of NF‐κB p65 in the nucleus was markedly increased after exposure to co‐treatment with LPS and ox‐LDL, and this response was significantly inhibited by PEITC.

**FIGURE 7:**
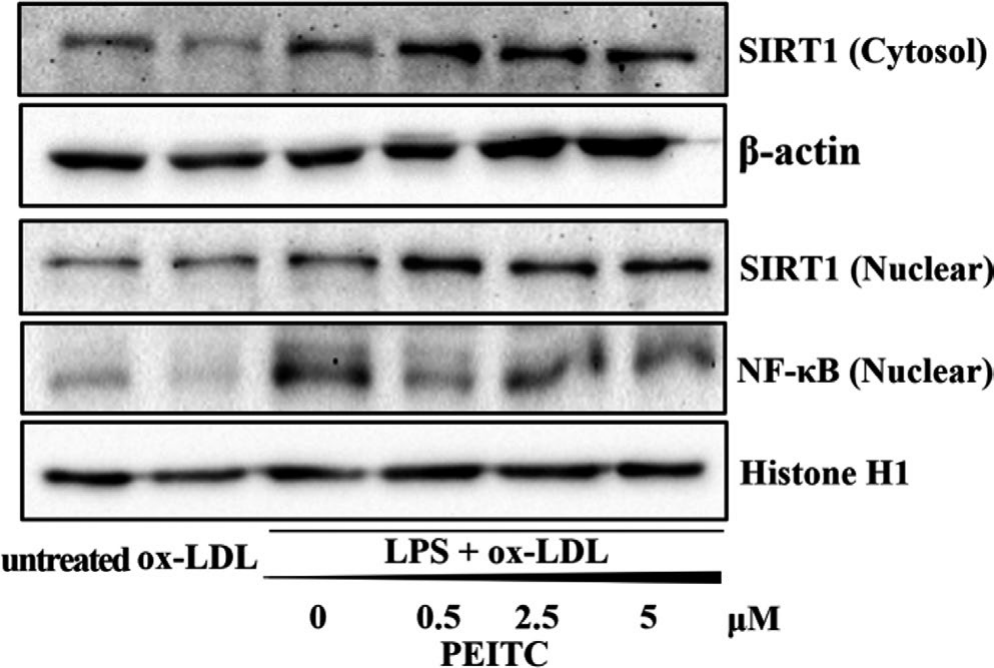
Effect of PEITC on the expression of SIRT1 and NF‐κB in THP‐1 foam cells. Immunoblotting was used to measure the protein levels of SIRT1 in cytosol and nucleus, NF‐κB in nucleus. β‐actin and Histone H1 were used as an internal control

SIRT1 blocks activation of the NF‐κB signaling pathway, which induces inflammation (Kauppinen et al., 2013). Therefore, we examined whether PEITC stimulates SIRT1 expression in the nucleus and cytosol by using immunoblotting (Figure 7). The results indicate that treatment with PEITC reduces inflammation in foam cells. PEITC treatment of foam cells upregulated the SIRT1 in nucleus. In addition, NF‐κB was downregulated by PEITC.

## 4 DISCUSSION

We investigated the effect and mechanism of atherosclerosis prevention using PEITC on THP‐1 derived foam cells. Lipid accumulation, foam cell formation, and inflammation are recognized as major features of atherosclerosis (Weber & Noels, 2011). In this study, we found that THP‐1‐derived macrophages treated with LPS and ox‐LDL together resulted in typical foam cell formation, and that PEITC effectively inhibited foam cell formation. Many reports have shown that hypercholesterolemia is a major risk factor for atherosclerosis, leading to the uptake of ox‐LDL by macrophages and foam cell formation (Gao & Liu, 2017; Gofman & Lindgren, 1950; Linton et al., 2000). ox‐LDL is involved in foam cell formation and triggers proatherogenic events such as overexpression of adhesion molecules and cytokines involved in the inflammatory process (Draude et al., 1999), cell proliferation, and apoptosis (Di Pietro et al., 2016). A previous study reported that LPS treatment in human macrophage‐derived foam cells with ox‐LDL suppressed cholesterol efflux and increased inflammation (Pennings et al., 2006; Wang et al., 2019, 2020).

In most studies, foam cells were formed when the ox‐LDL concentration was 50 μg/mL or higher (van Tits et al., 2011; Zhang et al., 2015). However, in our study, we confirmed that foam cells were formed when LPS was co‐treated with a lower concentration of ox‐LDL (20 μg/mL) than that used in most studies. Zhao *et al*. used ox‐LDL (50 μg/ml) and LPS (10 ng/ml) to induce foam cells in THP‐1 macrophage (Zhao et al., 2013). In another study, DONG et al. used ox‐LDL (50 μg/ml) and LPS 100 (ng/ml) to induce foam cells in THP‐1 macrophage (Dong et al., 2014). They determined the formation of foam cells by Oil Red O staining. It is also reported that LPS and ox‐LDL enhance the inflammatory response (Mikita et al., 2001; Rios et al., 2013).

Based on several studies, we designed a co‐treatment of LPS and ox‐LDL to induce the formation of foam cells. Therefore, we believe that our study is meaningful, as it suggested that co‐treatment with low concentrations of ox‐LDL and LPS accelerates foam cell formation in THP‐1 macrophages.

Recently, many studies have demonstrated that phytochemicals suppress ox‐LDL uptake by reducing SR‐A1, CD36, and LOX‐1 expression and attenuated foam cell formation in macrophages (Di Pietro et al., 2016; Ledda et al., 2016; Pirillo et al., 2013). For instance, protocatechuic acid, kaempferol, and quercetin have been reported to attenuate the formation of macrophage foam cells by downregulating CD36 expression and upregulating ABCA1 expression (Lee et al., 2013; Li et al., 2013).

We suggest that PEITC has a potential protective capacity against atherosclerosis by inhibiting total cholesterol influx and intracellular lipid accumulation. In our study, we observed that: (a) PEITC at ≥0.5 μM inhibited SR‐A1, CD36, and LOX‐1 expression, accounting for foam cell formation and cellular lipid accumulation in THP‐1 foam cells. (b) ox‐LDL and LPS treatment enhanced ABCA1 expression, which was further promoted at the transcriptional and protein levels by ≥0.5 μM PEITC. (c) PEITC promoted cholesterol efflux from lipid‐laden foam cells via PPARγ/LXR‐α/ABCA1 signaling. (d) PEITC also reduced pro‐inflammatory cytokines and transcription factors.

Macrophages internalize (uptake) ox‐LDL by SR‐A1, CD36, and LOX‐1 (Ooi et al., 2017). SR‐A and CD36 are expressed in human atherosclerotic lesions (Mäkinen et al., 2010) and have been shown to contribute to the uptake of modified LDL (Kunjathoor et al., 2002). In an in vivo study, dietary intake of walnut showed a protective effect on atherosclerosis with a decrease in CD36 expression in apoE‐/‐ mice (Crespo et al., 2008). Consistent with previous studies, we observed that PEITC moderated ox‐LDL by reducing SR‐A1, CD36, and LOX‐1 expression at the transcriptional level and attenuated foam cell formation full of cholesterol in the cytoplasm of macrophages. Furthermore, as evidenced by Oil Red O staining, in ox‐LDL and LPS co‐treatment, the typical formation of foam cells was observed. PEITC effectively suppressed foam cell formation induced by co‐treatment with ox‐LDL and LPS. Thus, we suggest that PEITC may inhibit foam cell formation by regulating lipid accumulation.

ABCA1, a key factor in cholesterol homeostasis, mediates the efflux of cellular free cholesterol and phospholipids to an extracellular acceptor, apoA‐I, to form nascent HDL (S. Wang & Smith, 2014). In this study, our data provide a new mechanism for the protective effect of PEITC on the development of atherosclerosis. We found that PEITC promotes cholesterol efflux in ox‐LDL‐loaded macrophages via regulation of ABCA1/LXR‐α/PPARγ expression. According to Lee et al., quercetin intake may contribute to lowering the risk of atherosclerosis by increasing the expression of PPARγ, LXR‐α, and ABCA1 genes and cholesterol efflux from THP‐1 macrophages (Lee et al., 2013). In agreement with this study, we suggest that PEITC enhances cholesterol efflux in ox‐LDL‐loaded macrophages by further inducing ABCA1/LXR‐α/PPARγ. Thus, we suggest that PEITC exerts anti‐atherosclerotic effects by suppressing foam formation of lipid deposition in THP‐1‐derived macrophages and by promoting the export of cholesterol from lipid‐laden macrophages to reverse cholesterol transport.

Evidence has shown that inflammation is a risk factor in the process of atherosclerosis (Jones et al., 2015). We observed that PEITC has anti‐inflammatory activities that inhibit LPS‐induced microglial activation via regulation of the SIRT1/NF‐κB pathway and thereby protects THP‐1 cells. After translocation into the nucleus, NF‐κB subunits, including p65 and p50, can be post‐translationally regulated by reversible acetylation. SIRT1 modulates the NF‐κB pathway via deacetylation of p65 at Lys310, thereby reducing inflammatory responses mediated by NF‐κB (Yang et al., 2012). Currently, some phytochemical compounds have been identified as having the ability to increase SIRT1 expression and activity, such as resveratrol and catechin (Cheng et al., 2019; Tian et al., 2016). In our study, co‐treatment with LPS and ox‐LDL significantly increased the levels of these cytokines, NF‐κB p65 (nuclear), and decreased SIRT1 (cytosol and nuclear) compared with the untreated cells using. However, these increases and decreases were significantly abrogated by PEITC. In addition, we observed that PEITC reduced IL‐6 and TNF‐α cytokine levels compared to co‐treatments with LPS and ox‐LDL. According to Mitsiogianni *et al*., one of the ITCs, sulforaphane has anti‐inflammatory effects in LPS‐stimulated Nrf2‐/‐macrophages, in which the absence of Nrf2 caused an increase in pro‐inflammatory markers, such as IL‐6 and TNF‐α (Mitsiogianni et al., 2019). These findings indicate that PEITC suppresses inflammatory transcription factor expression in the nucleus of foam cells and increases SIRT1 expression. Consequently, PEITC may reduce inflammation in the treatment of cardiovascular disease. However, we believe that further studies, including multiple cell lines and animal experiments, would be needed to discuss the application of the clinical environment.

In summary, our present study showed that PEITC could play a protective role in the early stage of atherosclerosis progression by promoting cholesterol efflux from lipid‐laden macrophages, reducing lipid accumulation, and modulating the SIRT1‐NF‐κB signaling pathway. Taken together, our findings reveal the mechanisms underlying atherosclerosis and provide a new therapeutic agent for the prevention and treatment of atherosclerosis.

## CONFLICT OF INTEREST

The authors declare that they do not have any conflict of interest.

## ETHICAL APPROVAL

This study does not involve any studies with human or animal subjects.
